# Correction: A segment of *Triticum timopheevii* chromosome 3G confers type II *Fusarium* head blight resistance and reduces DON accumulation in wheat

**DOI:** 10.3389/fpls.2026.1830108

**Published:** 2026-04-09

**Authors:** Andrew Steed, Surbhi Grewal, Roshani Badgami, Julie King, Ian P King, Paul Nicholson

**Affiliations:** 1 Department of Crop Genetics, John Innes Centre, Norwich, United Kingdom; 2 Nottingham Wheat Research Centre, School of Biosciences, University of Nottingham, Loughborough, United Kingdom

**Keywords:** breeding, *Fusarium* head blight, introgression, plant pathology, wheat

In the published article there was an omission in the **Funding** statement. The funders Research Ireland (RI), Northern Ireland’s Department of Agriculture, Environment and Rural Affairs (DAERA), UK Research and Innovation (UKRI) via the International Science Partnerships Fund (ISPF) under Grant numbers 22/CC/11147 (RI and DAERA) and BB/Y012909/1 (UKRI) at the Co-Centre for Sustainable Food Systems to P. Nicholson and R. Badgami were erroneously omitted.

The **Funding** statement should read:

“The author(s) declared financial support was received for this work and/or its publication. This work was supported by the United Kingdom Biotechnology and Biological Sciences Research Council through the Designing Future Wheat [BB/P016855/1] and Delivering Sustainable Wheat [BB/X011003/1] Institute Strategic Programmes. This publication has emanated from research conducted with the financial support of Research Ireland (RI), Northern Ireland’s Department of Agriculture, Environment and Rural Affairs (DAERA), UK Research and Innovation (UKRI) via the International Science Partnerships Fund (ISPF) under Grant numbers 22/CC/11147 (RI and DAERA) and BB/Y012909/1 (UKRI) at the Co-Centre for Sustainable Food Systems.”

